# Premarital Preventive Examinations and Access to Genetic Counselling in the South-Central Region of Bulgaria: Implications for Preconception and Child Health

**DOI:** 10.3390/healthcare14162524

**Published:** 2026-08-13

**Authors:** Eleonora Hristova-Atanasova, Teodora Gencheva, Elitsa Gyokova, Georgi Iskrov, Rumen Stefanov

**Affiliations:** 1 Department of Social Medicine and Public Health, Faculty of Public Health, Medical University of Plovdiv, 4002 Plovdiv, Bulgaria; georgi.iskrov@mu-plovdiv.bg (G.I.); rumen.stefanov@mu-plovdiv.bg (R.S.); 2 Faculty of Medicine, Medical University of Plovdiv, 4002 Plovdiv, Bulgaria; 20101026@mu-plovdiv.bg; 3 Department of Obstetrics and Gynecology, Faculty of Medicine, Medical University—Pleven, 5800 Pleven, Bulgaria; elitca.gaokova@mu-pleven.bg; 4 University Hospital “Saint Marina”—Pleven, 5800 Pleven, Bulgaria

**Keywords:** preconception care, reproductive health, genetic counselling, premarital screening, child health, health disparities, primary care, public health prevention

## Abstract

**Background:** Premarital medical certification is required before civil marriage in Bulgaria, but little is known about how newly married couples perceive this process within the broader framework of preconception care and genetic counselling. This study examined couples’ perceptions of premarital preventive examinations, reported receipt of genetic counselling, preferred counselling providers, and associated sociodemographic factors. **Methods:** A cross-sectional online survey was conducted between 2023 and 2025 among a convenience sample of 623 newly married heterosexual couples residing in the South-Central Region of Bulgaria. Each couple jointly completed a study-specific questionnaire assessing perceptions of premarital medical examinations, sources of preconception information, preferred counselling providers, and reported receipt of genetic counselling. Two multivariable binary logistic regression models were fitted to examine factors independently associated with reported receipt of genetic counselling and affirmative support for the necessity of premarital medical examination. **Results:** Overall, 77.8% of couples considered the premarital medical examination necessary, whereas 57.1% perceived it as primarily formal and 38.5% reported having received genetic counselling. Family (90.6%) and school (63.8%) were identified as responsible sources of preconception information more often than general practitioners (19.6%). In the adjusted model, each additional year of female age was associated with lower odds of reported genetic counselling receipt (aOR = 0.929, 95% CI: 0.900–0.959, *p* < 0.001). Bulgarian ethnicity was also associated with lower odds of reported counselling receipt compared with non-Bulgarian ethnicity (aOR = 0.499, 95% CI: 0.291–0.856, *p* = 0.012). Female age (aOR = 1.043, 95% CI: 1.006–1.081, *p* = 0.021) and employment (aOR = 1.847, 95% CI: 1.109–3.076, *p* = 0.018) were associated with affirmative support for the necessity of premarital medical examination. **Conclusions:** The findings describe perceptions and reported experiences within a regional online convenience sample and should not be interpreted as causal evidence or as a direct assessment of healthcare-system performance. They indicate areas warranting further evaluation, including the organisation of genetic counselling, the role of primary care, and equitable access to comprehensive preconception services.

## 1. Introduction

Premarital medical examinations represent an established public health initiative aimed at detecting health issues that could substantially affect reproductive outcomes. In Bulgaria, these examinations have been legally required for several decades and are a prerequisite for civil marriage. However, their formal preventive scope focuses mainly on selected infectious diseases and provides limited systematic assessment of non-communicable and genetic reproductive risks [1,2]. The existing regulatory framework does not mandate comprehensive assessment of chronic diseases, nutritional status, vaccination status, or genetic carrier status. Consequently, the formally defined scope of these examinations is narrower than that recommended in contemporary approaches to reproductive risk assessment [1,3]. While genetic counselling and genetic testing for hereditary conditions are accessible in Bulgaria, their incorporation into the premarital preventive examination framework lacks standardisation and continues to be opportunistic [2]. Use of genetic services appears limited, and out-of-pocket payment for many consultations and tests may create a financial barrier [2,4]. Unlike nations that have established comprehensive screening programmes for severe genetic conditions like thalassaemia, Bulgaria does not have structured initiatives for genetic screening aimed at the broader population or couples preparing for parenthood [3]. Accordingly, the potential contribution of compulsory premarital medical examinations to the prevention of genetic and non-infectious reproductive risks at the population level may be restricted. Despite broad international consensus on the essential components of preconception care (PCC), similar challenges arise at the European level during its implementation. Key recommendations emphasise that PCC should include a comprehensive personal and familial medical history, the assessment of genetic risks, advice on lifestyle and environmental influences, and a medication review [5]. Nonetheless, European health systems do not consistently apply these components, leading to significant variations in protocols, provider responsibilities, and service accessibility [5,6,7]. In the Netherlands, fewer than 50% of organisations that provide birth care use a formal protocol for PCC, resulting in large variations in the content and quality of consultations [6]. Uptake of PCC in Italy is low, with limited public awareness and limited involvement of healthcare professionals [7]. While policy frameworks and guidelines are available in the United Kingdom and Ireland [8], implementation is hampered by fragmented care pathways, lack of professional awareness and a focus on women, with less focus on male preconception health. Financial and systemic barriers are significant obstacles to accessing preventive and genetic services in both the European and Bulgarian contexts. Financial unaffordability remains a prevalent obstacle to healthcare access in Bulgaria, disproportionately impacting those with lower income, limited educational backgrounds, and rural living conditions, resulting in unmet healthcare needs [4,9]. These barriers may be associated with reduced access to genetic counselling and testing and could contribute to inequalities in the use of preventive services [4,10]. The absence of broadly implemented cascade screening and organised genetic risk-assessment initiatives may limit opportunities for systematic prevention. Available evidence also suggests that financial resources may influence access to genetic services [2,3]. In Bulgaria, general practitioners (GPs) are not obligated to provide comprehensive preconception or genetic counselling during the premarital medical examination, despite their pivotal role in primary care. Previous research suggests that limited training in medical genetics, the absence of standardised protocols, high workloads, and restricted consultation time may constrain the capacity of primary care providers to identify genetic and reproductive risks [11,12]. Primary care providers may have limited confidence, resources, and institutional support for initiating genetic risk assessment or making appropriate referrals. These circumstances may contribute to differences in access to genetic services, particularly in resource-constrained healthcare settings, including parts of Eastern Europe [12,13,14]. Collectively, the available evidence suggests that the potential preventive contribution of Bulgaria’s premarital medical examination system to non-infectious and genetic reproductive risk assessment may be influenced by systemic, financial, and educational barriers. The absence of compulsory genetic counselling, standardised protocols, and established mechanisms for equitable access may limit the extent to which this regulatory measure contributes to reproductive health prevention.

Despite the growing body of European literature on preconception care and the existence of mandatory premarital preventive examinations in Bulgaria, evidence integrating these two areas remains scarce. Previous studies have generally examined preconception care implementation, healthcare utilisation, or genetic services separately, whereas little is known about how mandatory premarital medical examinations are perceived as part of the broader preconception care framework. Furthermore, no previous Bulgarian study has simultaneously explored couples’ perceptions of mandatory premarital medical examinations, access to genetic counselling, and the perceived roles of different healthcare providers in delivering preconception care.

Within this conceptual framework, mandatory premarital preventive examinations are considered an entry point to preconception care, while access to genetic counselling and healthcare provider involvement represent key components of equitable preventive healthcare. Therefore, the present study aimed to evaluate couples’ perceptions of mandatory premarital preventive examinations, assess reported receipt of genetic counselling, explore associated sociodemographic factors, and identify patterns that may inform further evaluation of the organisation and accessibility of preconception care in Bulgaria.

## 2. Materials and Methods

### 2.1. Study Design and Setting

A cross-sectional online survey was conducted between 2023 and 2025 among newly married couples residing in the South-Central Region of Bulgaria. The study was coordinated by the Department of Social Medicine and Public Health, Faculty of Public Health, Medical University of Plovdiv.

The South-Central Region accounts for approximately one fifth of Bulgaria’s population and includes both urban and rural settlements. The study was designed to explore perceptions and reported experiences related to premarital preventive examinations and preconception care within this regional context. It was not designed to generate nationally representative prevalence estimates.

### 2.2. Participants and Eligibility Criteria

Eligible participants were heterosexual couples who had entered into a legally registered civil marriage no more than 12 months before completing the questionnaire. The 12-month eligibility interval was selected to limit, although not eliminate, potential recall bias regarding the premarital preventive examination.

The inclusion criteria were: (1) electronic informed consent provided by both partners; (2) civil marriage registered no more than 12 months before participation; (3) residence in the South-Central Region of Bulgaria; (4) at least one partner aged 18–49 years; and (5) joint completion of the shared couple-level questionnaire items.

Couples were excluded if either partner was a healthcare professional or if the couple reported a previous adverse pregnancy outcome. These criteria were applied to reduce the potential influence of professional healthcare knowledge or previous adverse reproductive experiences on perceptions of premarital medical examinations and preconception care. However, the possible implications of these exclusions for selection bias and generalisability are acknowledged in the Limitations section.

### 2.3. Data Collection and Recruitment

Data were collected using an anonymous, self-administered online questionnaire administered through Google Forms. Participation was voluntary, and no financial or other incentives were provided. No directly identifiable personal information was collected. Responses were stored in a password-protected database and analysed anonymously.

Participants were recruited through an open invitation published on the web-based educational platform *I Want a Healthy Child* (iskamdete.eu) and disseminated through its associated social media channels. Couples who accessed the invitation could review the study information and proceed to the questionnaire after both partners had provided electronic informed consent. Recruitment was not conducted through civil registries, healthcare facilities, or a predefined population sampling frame. The study therefore used a non-probability convenience sampling approach.

Because the invitation was openly accessible online, the number of individuals who viewed it or began the eligibility process could not be determined. Consequently, a conventional response rate could not be calculated. No formal a priori sample-size calculation was performed because the study was exploratory and recruitment was based on an open invitation without a predefined sampling frame. The final analytical sample comprised 623 eligible couples.

The questionnaire contained both shared couple-level items and partner-specific items. Shared items assessed the couples’ perceptions and reported experiences concerning premarital preventive examinations, sources of preconception information, preferred counselling providers, and receipt of genetic counselling. Sociodemographic characteristics were recorded separately for women and men. The perceived necessity of genetic counselling was also recorded separately for each partner. Accordingly, shared outcomes were analysed at the couple level, whereas partner-specific variables were analysed separately for women and men. This distinction was maintained throughout the statistical analysis and reporting of the results.

### 2.4. Questionnaire Instrument

A study-specific structured questionnaire was developed in Bulgarian by the research team. The instrument was not adapted directly from a single previously validated questionnaire. It was developed following a review of the relevant literature, current international recommendations on preconception care, Bulgarian regulations concerning mandatory premarital preventive examinations, and the objectives of the present study. A purpose-designed instrument was used because the research team did not identify a validated Bulgarian questionnaire covering the combined topics examined in this study.

The questionnaire consisted of 26 closed-ended items, including single-choice and multiple-choice questions, and required approximately 20–25 min to complete. The items covered: (1) sociodemographic characteristics of each partner; (2) perceptions of mandatory premarital preventive examinations; (3) perceived sources of family-planning and preconception information; (4) preferred providers of active reproductive health counselling; (5) reported receipt of genetic counselling; and (6) attitudes towards the necessity of genetic counselling.

The categories used to evaluate the premarital preventive examination were defined as subjective assessments of the participants’ experience. “Formal” referred to an examination perceived primarily as an administrative requirement with little preventive content; “superficial” referred to a limited assessment without comprehensive counselling or risk evaluation; and “in-depth” referred to an examination perceived as comprehensive and preventive in nature. These categories were not intended to represent externally verified indicators of healthcare quality.

The content, relevance, clarity, and comprehensiveness of the questionnaire items were reviewed by specialists in public health, reproductive medicine, and medical genetics. Face validity and comprehensibility were subsequently assessed through pilot testing involving 10 members of the target population. Based on their feedback, minor wording and clarity revisions were made before the main survey.

Formal construct validation, test–retest reliability assessment, and internal consistency analysis were not performed. Cronbach’s alpha was not calculated because the questionnaire items assessed distinct factual experiences, preferences, and descriptive perceptions and were not designed to form a unidimensional psychometric scale or composite score. This absence of formal psychometric validation is acknowledged as a limitation of the study.

### 2.5. Ethical Considerations

The study was anonymous and non-interventional and did not involve the collection of names, personal identification numbers, contact details, or other direct identifiers. The questionnaire collected self-reported information on premarital examinations, reproductive experiences, and genetic counselling. Electronic informed consent was obtained from both partners before they accessed and completed the questionnaire. Data were collected and processed in accordance with Regulation (EU) 2016/679 (General Data Protection Regulation). The study was conducted within the scientific and ethical scope of the project “Genome-Wide DNA Analysis of Pathogenic Copy Number Variations”, approved by the Ethics Committee for Research Activities at Medical University–Pleven under Decision No. 848 of 18 June 2025.

### 2.6. Statistical Analysis

Statistical analyses were performed using IBM SPSS Statistics for Windows, version 26.0 (IBM Corp., Armonk, NY, USA). Figures were prepared using Microsoft 365, version 2404 (Build 17531.20152). Continuous variables were summarised as means and standard deviations (SD), whereas categorical variables are reported as frequencies and percentages, n (%).

The couple was the primary observational unit for shared questionnaire responses concerning perceptions, attitudes, and reported experiences. Sociodemographic characteristics were recorded separately for women and men and were analysed as partner-specific explanatory variables in relation to the shared couple-level outcomes. Items answered individually by each partner, including the perceived necessity of genetic counselling, were analysed separately for women and men.

The item concerning perceived sources of preconception information permitted multiple responses and was defined in SPSS as a multiple-dichotomy response set. Associations between sociodemographic characteristics and the overall multiple-response pattern were assessed using the chi-square significance test for multiple-response sets available in IBM SPSS Custom Tables. Empty sociodemographic categories containing no observations were excluded from the analysis. Post hoc comparisons of column proportions were performed using Bonferroni adjustment within each subtable.

Associations between other categorical sociodemographic characteristics and categorical study outcomes were examined using Pearson’s chi-square test. Where more than 20% of cells had expected counts below five, Monte Carlo estimates of the exact two-sided *p*-value were calculated using 10,000 sampled tables and were used for statistical inference. Pearson’s correlation coefficient was used to assess the association between female and male age. The bivariate analyses were considered secondary and exploratory. Because no study-wide adjustment was applied across all omnibus tests, individual nominal *p*-values were interpreted cautiously and were not used alone to support the principal conclusions.

Two multivariable binary logistic regression models were fitted to examine factors independently associated with: (i) reported receipt of premarital genetic counselling and (ii) affirmative support for the necessity of premarital medical examination. In the first model, receipt of premarital genetic counselling was coded as 1 for “yes” and 0 for “no”. In the second model, perceived necessity of premarital medical examination was coded as 1 for “yes”, whereas “no” and “cannot judge” were combined and coded as 0, thereby distinguishing affirmative support from the absence of affirmative support.

Female age was entered as a continuous variable. Educational attainment was dichotomised as higher education versus secondary education or lower; employment status as employed versus not employed; place of residence as urban versus rural; and ethnicity as Bulgarian versus non-Bulgarian. All explanatory variables were entered simultaneously using simultaneous entry (the Enter method in SPSS).

A single consistent set of female sociodemographic characteristics was used in both models to maintain analytical consistency and model parsimony and to avoid the simultaneous inclusion of parallel and potentially correlated characteristics from both partners. Male age was not included because it was strongly correlated with female age (Pearson’s r = 0.847), which could increase the risk of multicollinearity. The implications of excluding male sociodemographic characteristics from the multivariable models are acknowledged in the Limitations section.

Adjusted odds ratios (aORs) with 95% confidence intervals (95% CIs) were reported. Overall model significance was assessed using the omnibus likelihood-ratio chi-square test, model calibration was evaluated using the Hosmer–Lemeshow goodness-of-fit test, and model explanatory capacity was summarised using Nagelkerke’s pseudo-R^2^. All statistical tests were two-sided, and *p* < 0.05 was considered statistically significant. Values generated by SPSS as *p* = 0.000 were reported as *p* < 0.001. Given the cross-sectional design, all results were interpreted as associations and not as evidence of causal relationships.

## 3. Results

### 3.1. Sociodemographic Characteristics

The study included 623 married couples. Unless otherwise stated, outcome variables presented in the following sections reflect responses jointly provided by each couple, whereas women’s and men’s sociodemographic characteristics were analysed separately as explanatory variables. Overall, participants were predominantly of Bulgarian ethnicity, highly educated, and urban residents. Women were younger than men on average and were more frequently students, whereas men reported higher employment rates. Within-couple concordance was strongest for ethnicity and age group, while agreement for education and employment status was lower (Table 1).

### 3.2. Responsibility for the Provision of Family Planning and PCC Information

Participants were asked to identify which stakeholders should be responsible for providing information on family planning and preconception care. Multiple responses were permitted, and all responses to this item represented shared couple-level perceptions. Overall, the family was the most frequently selected source of preconception information (90.6%, n = 565), followed by school (63.8%, n = 398), whereas the GP was selected by fewer than one fifth of couples (19.6%, n = 122) (Table 2).

Significant differences in the overall multiple-response pattern were observed according to female ethnicity, χ^2^(6) = 17.99, *p* = 0.006; female employment status, χ^2^(6) = 25.58, *p* < 0.001; female educational attainment, χ^2^(6) = 45.51, *p* < 0.001; male educational attainment, χ^2^(6) = 26.01, *p* < 0.001; and male age group, χ^2^(3) = 10.70, *p* = 0.013. No statistically significant differences were identified according to female age group, male ethnicity, male employment status, or place of residence.

Descriptively, the GP was more frequently selected among couples in which the woman was studying or had primary or lower education. School was also selected most frequently among couples in which the woman was studying. The family remained the most frequently selected source across most sociodemographic subgroups, particularly among couples in which either partner had secondary or higher education. Among male age groups, couples with men aged ≤ 30 years more frequently selected both the GP and school than couples with men aged > 30 years.

Because these bivariate analyses were exploratory and some sociodemographic subgroups were relatively small, the observed subgroup patterns should be interpreted cautiously. Across the full sample, the family remained the dominant perceived source of preconception information, followed by school, whereas the GP was selected considerably less frequently.

### 3.3. Preferred Provider of Active Counselling on Family Planning and Reproductive Health

When asked who should actively provide counselling on family planning and reproductive health, most couples preferred an obstetrician–gynaecologist (OB-GYN) (72.1%, n = 449), whereas fewer selected a GP (24.2%, n = 151) or another healthcare professional (3.7%, n = 23).

Significant differences in provider preference were observed based on the ethnicity of both women (χ^2^ = 16.854, *p* = 0.002) and men (χ^2^ = 14.346, *p* = 0.006), as well as women’s educational attainment (χ^2^ = 11.315, *p* = 0.023) and men’s employment status (χ^2^ = 14.943, *p* = 0.005). No statistically significant associations were found for age group, women’s employment status, men’s educational attainment, or place of residence (*p* > 0.05).

**Figure 1:**
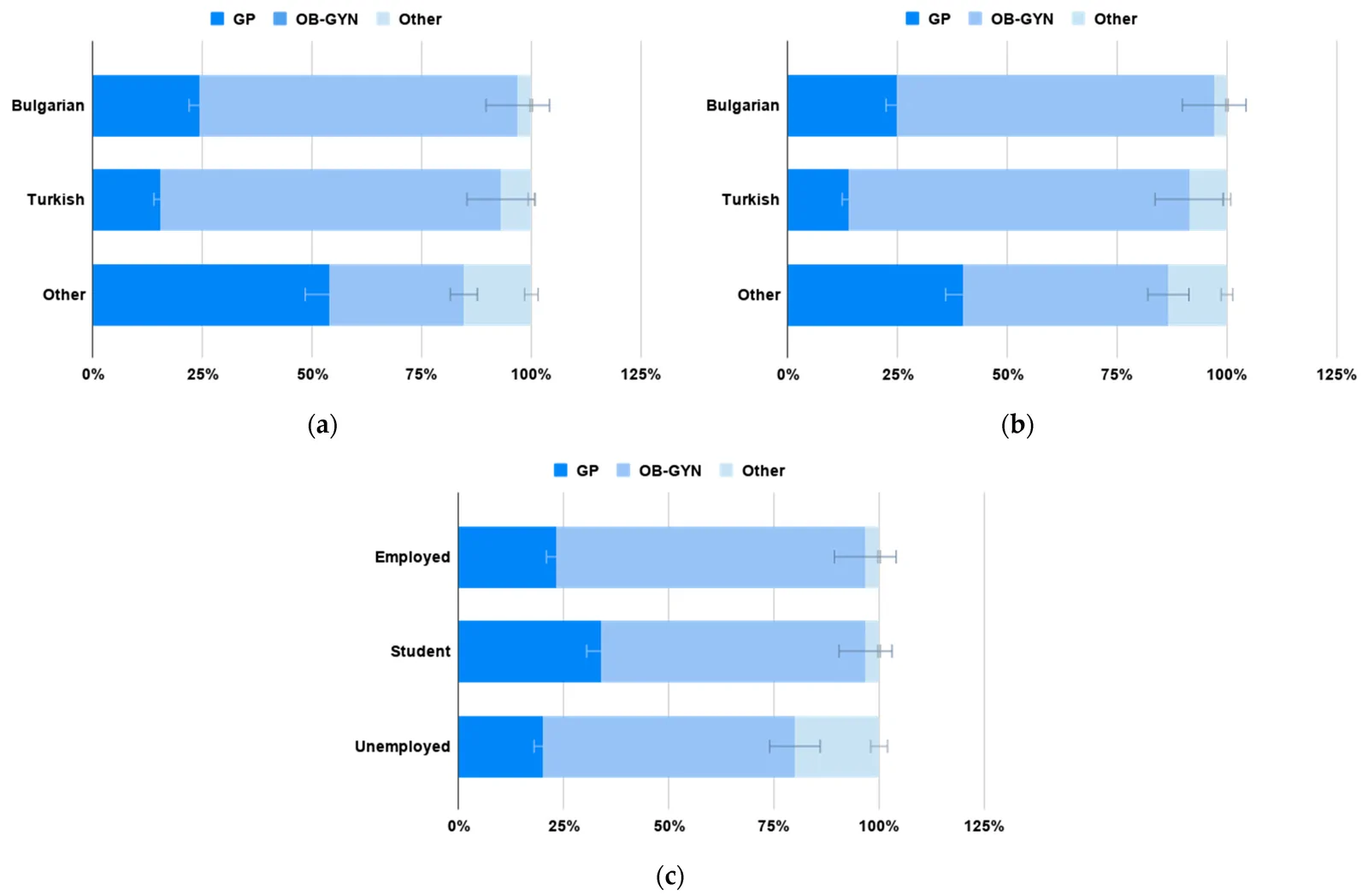
Preferred provider of active counselling on family planning and reproductive health according to selected sociodemographic characteristics: (**a**) female ethnicity; (**b**) male ethnicity; and (**c**) male employment status. Each stacked bar represents the percentage distribution of couples preferring a general practitioner (GP), an obstetrician–gynaecologist (OB-GYN), or another healthcare professional within the respective sociodemographic subgroup.

Across all sociodemographic groups, the OB-GYN remained the predominant preferred counselling provider. Selected distributions according to female ethnicity, male ethnicity, and male employment status are presented in Figure 1. The variation mainly reflected differences in the proportions preferring a GP or another healthcare professional.

A statistically significant association was observed between the preferred counselling provider and the overall multiple-response pattern of perceived preconception information sources, χ^2^ = 43.554, *p* < 0.001. Couples who preferred a GP as their active counselling provider more frequently identified the GP as a responsible source of preconception information. Nevertheless, the family remained the most frequently selected source across all provider-preference groups, while school was also commonly identified.

### 3.4. Attitudes Toward the Necessity of Premarital Medical Examination

Overall, 77.8% (n = 485) of couples considered the premarital medical examination necessary, whereas 11.7% (n = 73) considered it unnecessary and 10.4% (n = 65) were undecided.

**Figure 2:**
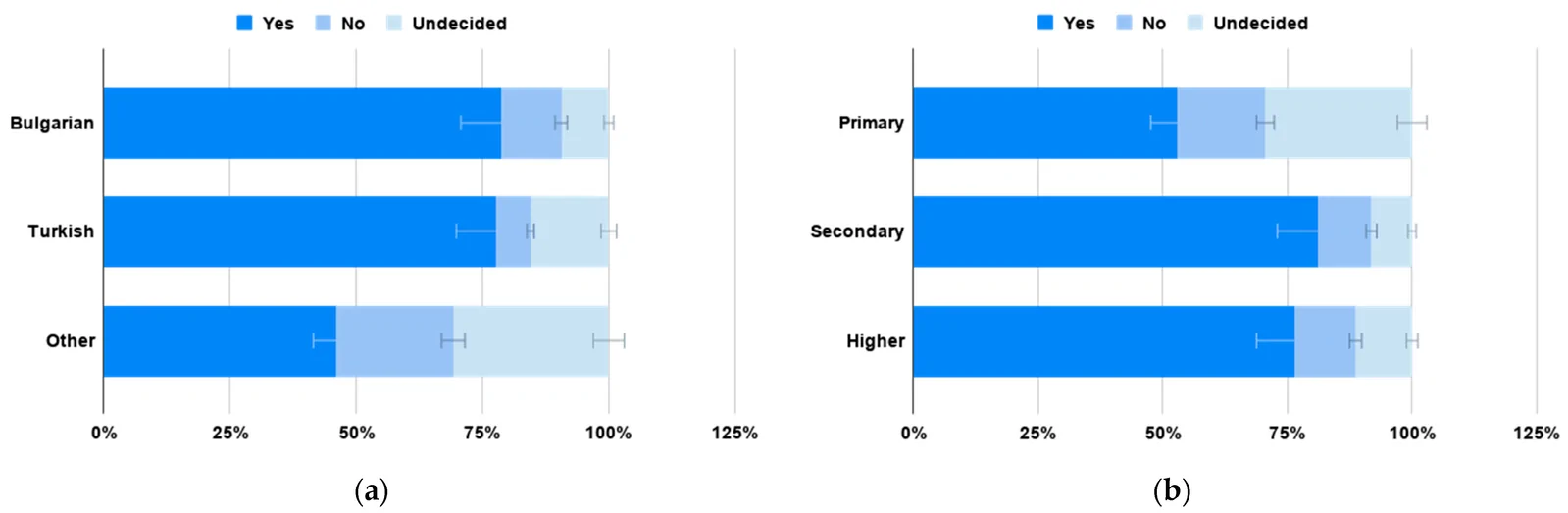
Attitudes towards the necessity of premarital medical examination according to: (**a**) female ethnicity and (**b**) male educational attainment. Each stacked bar represents the percentage distribution of couples who considered the premarital medical examination necessary, unnecessary, or were undecided within the respective sociodemographic subgroup.

Attitudes towards the necessity of premarital medical examination were significantly associated with female ethnicity, χ^2^(4) = 11.462, *p* = 0.022, and male educational attainment, χ^2^(4) = 10.068, *p* = 0.039. Affirmative support was descriptively lower among couples in which the woman belonged to the “Other” ethnic category; however, this subgroup was small and the pattern should be interpreted cautiously. Support was also lower among couples in which the man had primary or lower educational attainment than among those in which he had secondary or higher education (Figure 2).

No statistically significant associations were identified for male ethnicity or for the age group or employment status of either partner (all *p* > 0.05). No statistically significant association was observed between attitudes toward premarital medical examination and perceived sources of preconception information (*p* > 0.05).

### 3.5. Evaluation of the Premarital Preventive Examination and Genetic Counselling

Participants classified their overall experience of the premarital medical examination using predefined perception categories included in the questionnaire. “Formal” referred to an examination perceived primarily as an administrative requirement, “superficial” indicated limited clinical assessment without comprehensive counselling, whereas “in-depth” described an examination perceived as comprehensive and preventive in nature. Most couples described the premarital medical examination as formal (57.1%, n = 356); 27.3% (n = 170) described it as superficial, and 15.6% (n = 97) as in-depth.

Evaluation of the premarital medical examination was significantly associated with women’s employment status, χ^2^(4) = 32.624, *p* < 0.001; women’s educational attainment, χ^2^(4) = 21.747, Monte Carlo *p* < 0.001; women’s age group, χ^2^(2) = 12.395, *p* = 0.002; and men’s age group, χ^2^(2) = 18.674, *p* < 0.001. Older participants and couples in which the woman had higher educational attainment more frequently characterised the examination as formal. No statistically significant associations were observed according to women’s ethnicity, χ^2^(4) = 3.203, Monte Carlo *p* = 0.539; men’s ethnicity, χ^2^(4) = 7.472, Monte Carlo *p* = 0.108; men’s employment status, χ^2^(4) = 8.822, Monte Carlo *p* = 0.061; men’s educational attainment, χ^2^(4) = 6.530, Monte Carlo *p* = 0.158; or place of residence, χ^2^(2) = 0.013, *p* = 0.993 (Table 3). Overall, 38.5% (n = 240) of couples reported having received genetic counselling, whereas 61.5% (n = 383) had not. Receipt of consultation was significantly associated with the ethnicity of both women (χ^2^ = 14.974, *p* = 0.001) and men (χ^2^ = 16.642, *p* < 0.001); men’s educational attainment (χ^2^ = 11.689, *p* = 0.003); and age group among both women (χ^2^ = 27.008, *p* < 0.001) and men (χ^2^ = 17.681, *p* < 0.001). Consultation was reported more frequently among younger respondents and among participants belonging to minority ethnic groups. No significant associations were identified for employment status (*p* > 0.05).

Most women (86.5%, n = 539) and men (78.0%, n = 486) considered genetic counselling necessary. No significant sociodemographic differences were observed in women’s perceptions (*p* > 0.05). Among men, however, perceived necessity was associated with educational attainment (χ^2^ = 6.347, *p* = 0.042) and age group (χ^2^ = 5.221, *p* = 0.022), with stronger endorsement among younger and less educated respondents (Table 3).

### 3.6. Multivariable Logistic Regression Analysis

Two multivariable binary logistic regression models were fitted to examine factors independently associated with: (i) reported receipt of genetic counselling and (ii) affirmative support for the necessity of premarital medical examination. The results are presented in Table 4.

The first model, examining reported receipt of genetic counselling, was statistically significant, χ^2^(5) = 37.503, *p* < 0.001, with a Nagelkerke pseudo-R^2^ of 0.079. The Hosmer–Lemeshow test indicated adequate model calibration, χ^2^(8) = 6.153, *p* = 0.630. Each additional year of female age was associated with a 7.1% reduction in the adjusted odds of reported counselling receipt (aOR = 0.929, 95% CI: 0.900–0.959, *p* < 0.001). Bulgarian ethnicity was associated with lower adjusted odds of reported counselling receipt compared with non-Bulgarian ethnicity (aOR = 0.499, 95% CI: 0.291–0.856, *p* = 0.012). Educational attainment, employment status, and place of residence were not independently associated with the outcome.

The second model, examining affirmative support for the necessity of premarital medical examination, was also statistically significant, χ^2^(5) = 11.922, *p* = 0.036, although its explanatory capacity was modest (Nagelkerke pseudo-R^2^ = 0.029). The Hosmer–Lemeshow test indicated acceptable model calibration, χ^2^(8) = 7.674, *p* = 0.466. Each additional year of female age was associated with higher adjusted odds of affirmative support (aOR = 1.043, 95% CI: 1.006–1.081, *p* = 0.021). Employment was also independently associated with the outcome, with employed women having higher adjusted odds of affirmative support than women who were not employed (aOR = 1.847, 95% CI: 1.109–3.076, *p* = 0.018). Educational attainment, ethnicity, and place of residence were not independently associated with the outcome.

## 4. Discussion

### 4.1. Principal Findings in the European Context

The present study examined mandatory premarital preventive examinations as a potential entry point to broader preconception care from the perspective of newly married couples. Four principal findings emerged. First, although more than three quarters of couples considered the premarital medical examination necessary, most perceived their own examination as formal or superficial rather than comprehensive. Second, fewer than two fifths reported having received genetic counselling. Third, the family and school were much more frequently identified as responsible sources of preconception information than general practitioners, whereas most couples preferred active counselling from an obstetrician–gynaecologist. Fourth, the adjusted analyses identified female age and ethnicity as factors associated with reported receipt of genetic counselling, whereas female age and employment were associated with affirmative support for the necessity of premarital medical examination.

These findings should be considered within the heterogeneous European context of preconception care. A substantial proportion of women enter pregnancy with at least one potentially modifiable risk factor, while comparatively few seek professional preconception advice or make appropriate behavioural changes before conception [15]. This indicates an ongoing discrepancy between recognition of reproductive risks and the routine delivery and uptake of preventive care.

Although many European countries lack a comprehensive national approach to preconception care, selected elements have been incorporated into broader clinical guidance. In Scotland, for example, preconception risk assessment is included in recommendations concerning women of reproductive age with cardiovascular disease [16]. In the Netherlands, preconception care is generally delivered through individualised consultations by midwives and GPs; however, fewer than half of relevant practices reportedly use a formal protocol, and professional approaches to risk identification remain variable [6,17]. Intervention studies have demonstrated improvements in selected preconception behaviours following targeted counselling and community-based health-promotion initiatives [18,19], although comprehensive programmes covering all couples planning pregnancy remain limited despite initiatives intended to strengthen provider participation [20].

Similarly, evidence from Italy indicates relatively limited engagement with preconception consultations, although attendance has been associated with higher uptake of folic acid supplementation and vaccination [21]. In the United Kingdom and Ireland, preconception care is recognised as important, but its routine integration into general practice is constrained by limited time, resources, professional awareness, and patient engagement [22,23]. Differences in awareness between women and men have also been reported, supporting the need for educational approaches directed towards both partners [22]. Broader European assessments of reproductive healthcare services have likewise demonstrated substantial variation in service organisation, access, and professional responsibilities across countries [24].

Against this background, the present study contributes regional Bulgarian evidence by integrating couples’ perceptions of mandatory premarital med examinations, reported receipt of genetic counselling, and perceived responsibilities of healthcare providers. However, the study assessed perceptions and self-reported experiences rather than healthcare organisations directly. Its findings should therefore be interpreted as identifying areas for further evaluation rather than as demonstrating structural deficiencies in the healthcare system.

### 4.2. Premarital Preventive Examinations: Formalisation Versus Prevention

The findings indicate that premarital medical examinations were broadly accepted by the participating couples, but were frequently perceived as formal or administrative requirements rather than as comprehensive preventive interventions. This pattern is compatible with wider European evidence showing that preconception care may remain fragmented or incompletely integrated into routine healthcare in the absence of comprehensive national strategies and standardised protocols [15,16].

A notable discrepancy was observed between support for the examination and participants’ evaluation of their own experience. Although 77.8% considered the examination necessary, 57.1% described it as formal and a further 27.3% as superficial. Acceptance of the legal requirement therefore did not necessarily correspond to a perception that the examination included comprehensive risk assessment, counselling, or preventive follow-up.

Couples in which the woman had higher educational attainment more frequently characterised the examination as formal. This association may reflect differences in health literacy, expectations regarding preventive healthcare, previous contact with health services, or other unmeasured characteristics. These potential explanations were not directly assessed and should therefore be regarded as hypotheses rather than demonstrated mechanisms. Evidence from Italy, the United Kingdom, and Ireland similarly suggests that uptake and perceived relevance may depend on how clearly preconception care is incorporated into routine practice and communicated to prospective parents [21,22,23].

The multivariable analysis demonstrated that the factors associated with reported use of genetic counselling were not identical to those associated with attitudes towards premarital medical examination. Increasing female age was associated with lower adjusted odds of reported counselling receipt, but with higher adjusted odds of considering the premarital medical examination necessary. This apparent divergence may reflect age-related differences in healthcare-seeking behaviour, exposure to reproductive health information, reproductive intentions, or contact with preventive services. None of these possible mechanisms was measured directly.

Bulgarian ethnicity was associated with lower adjusted odds of reported counselling receipt compared with non-Bulgarian ethnicity. This unexpected finding should not be interpreted as evidence of systematically better or more equitable access among minority groups. The non-Bulgarian category combined heterogeneous ethnic groups, some individual subgroups were small, and the study did not assess referral indications, family history, previous reproductive experiences, service availability, or the circumstances in which counselling was received. Confirmation in larger and more diverse samples is therefore required.

Employment was independently associated with affirmative support for the necessity of premarital medical examination. This association may reflect differences in socioeconomic circumstances, social participation, healthcare access, health literacy, or previous experience with preventive services. These factors were not measured, and the association should not be interpreted causally.

The modest Nagelkerke pseudo-R^2^ values further indicate that the included sociodemographic characteristics accounted for only a limited proportion of the observed variation. Other factors, including family history, health literacy, reproductive intentions, previous healthcare experiences, financial circumstances, referral pathways, and service availability, may also be relevant. Several bivariate associations were attenuated after simultaneous adjustment for age, ethnicity, education, employment, and residence, although residual confounding by unmeasured variables cannot be excluded.

### 4.3. Educational Institutions, Families, and Healthcare Providers

The family was the most frequently identified source of preconception information, followed by school, whereas fewer than one fifth of couples identified the GP as responsible for providing such information. These findings reflect participants’ perceptions of responsibility and should not be interpreted as evidence regarding the accuracy, completeness, or effectiveness of information received from the respective sources.

Educational institutions are increasingly recognised as important settings for foundational reproductive and preconception health education. Recommendations have been made to strengthen curricula in order to prepare young people for future parenthood, although implementation remains inconsistent and may be limited by insufficient training and resources available to educators and youth workers [25]. The prominence of school in the present study suggests that couples view reproductive health education as a responsibility that begins before pregnancy planning or direct contact with reproductive healthcare services.

Families may also play an important role in transmitting reproductive health beliefs and knowledge and in collecting and communicating family medical and genetic history. A comprehensive family history obtained from both partners may support the identification of hereditary disorders, congenital anomalies, and potential indications for genetic counselling. When carrier status or increased genetic risk is identified, counselling may help couples understand inheritance patterns, reproductive options, and potential interventions, including preimplantation genetic testing [26].

Most couples nevertheless preferred active counselling from an obstetrician–gynaecologist, whereas approximately one quarter selected a GP. This preference may reflect the perceived clinical relevance and specialist expertise of obstetrician–gynaecologists in reproductive healthcare. In several European systems, however, GPs and midwives have an important role in the delivery of preconception care because of their accessibility and continuing relationships with patients, as illustrated by the Dutch model [6,17].

In England, preconception care may be available through general practice, but uptake remains limited and often depends on women actively seeking advice. Routine health-promotion activities may also take priority over dedicated preconception consultations, potentially reducing opportunities for focused risk assessment and counselling. Obstetrician–gynaecologists are more commonly involved when reproductive or medical problems arise or when specialist referral is required [27,28].

European expert recommendations emphasise that healthcare professionals caring for people of reproductive age should use appropriate opportunities to provide preconception advice or facilitate referral. Nevertheless, unclear professional responsibilities, limited training, time constraints, and insufficient resources continue to impede consistent implementation [29,30,31].

In the present study, couples who preferred a GP as their active counselling provider also more frequently identified the GP as a responsible source of preconception information. This association may reflect previous experience with, trust in, or expectations of primary care; however, these factors were not assessed. The results support further evaluation of GP training, practical guidance, and referral pathways linking primary care with specialised reproductive and genetic services.

### 4.4. Genetic Counselling and Equity of Access

Only 38.5% of couples reported having received genetic counselling. Because the questionnaire did not assess the indication, timing, content, duration, or provider of the counselling, this percentage should not be interpreted as a direct measure of service coverage or quality. It represents self-reported receipt of counselling that may have occurred in different clinical or reproductive contexts.

Reported counselling receipt was more frequent among couples with younger women and among those in which the woman was classified as non-Bulgarian. Educational differences were observed in the bivariate analyses, but female educational attainment was not independently associated with counselling receipt after adjustment. This attenuation suggests that part of the unadjusted association may have reflected relationships with age, employment, ethnicity, or other included characteristics.

The mechanisms underlying the observed associations cannot be determined from the present data. Possible explanations include differences in clinical indications, referral practices, family history, previous reproductive experiences, health literacy, service awareness, or contact with specialised healthcare. The study did not directly examine these factors.

The absence of a standardised approach to genetic counselling within premarital medical examinations may contribute to variation in how and when such services are accessed, although the present study did not evaluate service organisation directly. Previous evidence from Bulgaria indicates that genetic counselling is frequently sought following a specific clinical indication rather than as a routine component of preventive reproductive care [2]. Similarly, the absence of population-based carrier-screening programmes may limit opportunities for systematic identification of couples at increased risk of selected inherited disorders [32,33].

Financial and organisational factors may also influence access to genetic services. Out-of-pocket payments and informal healthcare expenditure have been associated with unmet healthcare needs and unequal service utilisation in Bulgaria [4,9,10]. Their specific role in the receipt of genetic counselling in the present sample was not assessed, and financial explanations should therefore not be inferred directly from the observed associations.

Taken together, the findings raise questions regarding the consistency and accessibility of genetic counselling within preconception care. Potential areas for future evaluation include clearer referral criteria, better coordination between primary care and specialised services, and the feasibility of publicly supported counselling or carrier-screening approaches where clinically and epidemiologically appropriate. The present data support further investigation of these options but do not establish their effectiveness, feasibility, or cost-effectiveness.

### 4.5. The Gender Dimension in Preconception Care

Contemporary preconception care frameworks increasingly recognise reproductive health as a shared responsibility of women and men, although research and service implementation remain predominantly focused on women. A scoping review found that only a small proportion of preconception health studies included or specifically addressed men, demonstrating a continuing gap in the evidence base [34].

Research from Ireland and the United Kingdom similarly indicates that, although both women and men recognise the relevance of preconception health, professional communication and service delivery remain more strongly directed towards women, while men’s roles are less clearly defined [22,35]. Broader reviews also identify limited awareness among both partners and emphasise the influence of cultural expectations and gender norms on engagement with preconception care. Partnership-oriented approaches have therefore been proposed as a means of promoting shared responsibility for reproductive health [5,36,37,38,39].

The present questionnaire combined shared couple-level outcomes with sociodemographic characteristics recorded separately for women and men. Partner-specific characteristics were consequently examined in relation to the same couple-level perceptions and experiences. This analytical approach did not constitute a direct comparison between women and men and did not test formal sex- or gender-based interaction effects.

Different patterns of association were observed in the partner-specific analyses. For example, women’s employment status and educational attainment were associated with evaluation of the premarital medical examination, whereas men’s educational attainment was associated with some individually reported attitudes. However, a statistically significant association in one partner-specific analysis and a non-significant association in another does not by itself demonstrate a difference between women and men. Because formal interaction analyses were not performed, these patterns should not be interpreted as confirmed gender differences.

The inclusion of both partners nevertheless supports the relevance of couple-oriented preconception care. Joint approaches may facilitate communication regarding family history, reproductive intentions, genetic risks, preventive behaviours, and available services. Future studies should collect individual-level outcomes from both partners and formally examine partner-specific effects and interactions to clarify how sex- and gender-related factors influence engagement with preconception care.

### 4.6. Strengths, Limitations and Implications for Public Health

The study has several strengths, including its relatively large sample of 623 couples, the inclusion of both partners, and the simultaneous assessment of premarital preventive examinations, reported receipt of genetic counselling, and perceived healthcare-provider responsibilities within a couple-based framework. Nevertheless, several limitations should be acknowledged.

First, the cross-sectional design precludes causal inference. Recruitment through an open invitation on a web-based educational platform resulted in a non-probability convenience sample and may have preferentially attracted individuals with greater interest in reproductive health, higher digital literacy, and easier internet access. The study population was predominantly urban and relatively highly educated, and the number of individuals exposed to the invitation was unknown; therefore, a conventional response rate could not be calculated. Consequently, the sample should not be considered representative of all newly married couples in the South-Central Region, and the findings should not be generalised to the entire Bulgarian population or to other regions without caution.

Second, the study relied on self-reported information. Although eligibility was restricted to couples married within the preceding 12 months, residual recall bias concerning the premarital medical examination and any counselling received cannot be excluded. Joint completion of the questionnaire may also have introduced social-desirability or within-couple consensus bias, because partners may have modified their responses in each other’s presence rather than expressing fully independent opinions.

The exclusion of healthcare professionals was intended to reduce the influence of specialised medical knowledge, while the exclusion of couples with previous adverse pregnancy outcomes was intended to limit the influence of prior reproductive experiences. However, these criteria may have introduced additional selection bias and limited the applicability of the findings to these groups.

Third, the study-specific questionnaire underwent expert review of its content and pilot assessment of face validity and comprehensibility but did not undergo formal construct validation, test–retest reliability assessment, or internal-consistency analysis. This may affect the reproducibility and measurement validity of the findings.

Several secondary bivariate comparisons were performed without a study-wide adjustment for multiplicity. Bonferroni adjustment was applied to post hoc comparisons within the multiple-response subtables, and Monte Carlo estimates of exact two-sided *p*-values were used where more than 20% of expected cell counts were below five. Nevertheless, some nominally significant findings may represent type I error. The bivariate results should therefore be regarded as exploratory and hypothesis-generating, particularly where subgroup sizes were small.

Fourth, the multivariable models used a single consistent set of female sociodemographic characteristics to maintain analytical consistency and model parsimony and to avoid the simultaneous inclusion of parallel and potentially correlated characteristics from both partners. Male age was excluded because of its strong correlation with female age. However, the exclusion of male sociodemographic characteristics from the models may have limited the assessment of couple-level determinants. Because the principal outcomes were largely shared couple-level responses, direct comparisons and interaction analyses between women and men were beyond the scope of the study design.

The relatively low Nagelkerke pseudo-R^2^ values also indicate that important unmeasured factors may contribute to the observed outcomes. These may include health literacy, family history, previous healthcare experiences, clinical indications for counselling, reproductive intentions, financial circumstances, referral pathways, and service availability.

Finally, the findings should not be interpreted as direct measures of healthcare-system performance or as evidence that organisational, financial, cultural, or provider-level factors caused the observed associations. Rather, they identify patterns warranting further investigation. Longitudinal, nationally representative, and mixed-methods research is needed to examine the mechanisms underlying these patterns.

From a public health perspective, the findings suggest potential priorities for future evaluation, including the development of standardised genetic counselling and referral pathways, strengthened GP training in preconception care and reproductive genetics, improved coordination with specialised services, and more accessible couple-oriented approaches that actively involve both partners. Publicly supported counselling and structured carrier-screening strategies may also warrant evaluation where appropriate, but their implementation should be informed by further evidence regarding clinical indications, feasibility, equity, and cost-effectiveness.

## 5. Conclusions

This study found that mandatory premarital medical examinations were widely supported among the participating newly married couples, although they were frequently perceived as formal or administrative procedures rather than as comprehensive preventive interventions. Reported receipt of genetic counselling was independently associated with female age and ethnicity, whereas affirmative support for the necessity of premarital medical examination was independently associated with female age and employment status. These associations should be interpreted cautiously and should not be regarded as evidence of causal relationships or as direct measures of healthcare-system performance.

The relatively low perceived responsibility attributed to primary care providers and the strong preference for specialist counselling suggest several practical priorities. These include the development of standardised protocols for genetic counselling within mandatory premarital medical examinations, strengthening general practitioners’ competencies in preconception care and reproductive genetics, improving referral pathways between primary care and specialised genetic services, and promoting equitable access to counselling through publicly supported preventive programmes.

Further nationally representative, longitudinal, and mixed-methods research is needed to examine the organisational, financial, cultural, and clinical factors underlying the observed patterns. Such evidence could support the development of evidence-based national policies integrating structured genetic counselling, stronger primary care involvement, equitable access to preventive genetic services, and gender-sensitive preconception care.

## Figures and Tables

**Table 1:** Sociodemographic characteristics of married couples.

Variable	Category	Female, n (%)	Male, n (%)	Female–Male Agreement Within Couples (%, κ)
Age group	≤30 years	399 (64.0)	331 (53.1)	82.6%; κ = 0.647
	>30 years	224 (36.0)	292 (46.9)	
Ethnicity	Bulgarian	552 (88.6)	550 (88.3)	96.5%; κ = 0.831
	Turkish	58 (9.3)	58 (9.3)	
	Other	13 (2.1)	15 (2.4)	
Educational attainment	Primary or lower	15 (2.4)	17 (2.7)	69.0%; κ = 0.387
	Secondary	188 (30.2)	270 (43.3)	
	Higher	420 (67.4)	336 (53.9)	
Employment	Employed	418 (67.1)	546 (87.6)	73.2%; κ = 0.300
	Studying	173 (27.8)	62 (10.0)	
	Unemployed	32 (5.1)	15 (2.4)	

Values are presented as n (%). Percentages were calculated separately for women and men. Female–male agreement within couples represents the observed within-couple concordance (diagonal/valid pairs) and κ denotes Cohen’s kappa. n = 623 valid couples.

**Table 2:** Perceived sources of preconception information according to sociodemographic characteristics.

Variable	Sex	Category	GP, % (n)	School, % (n)	Family, % (n)	χ^2^(df)	*p*
Age group	Woman	≤30 years	20.6 (82)	66.9 (267)	91.2 (364)	4.95 (3)	0.176
		>30 years	17.9 (40)	59.4 (133)	89.7 (201)		
	Man	≤30 years	23.0 (76)	68.3 (226)	91.2 (302)	10.70 (3)	0.013 *
		>30 years	15.8 (46)	59.6 (174)	90.1 (263)		
Educational attainment	Woman	Primary or lower	73.3 (11)	80.0 (12)	73.3 (11)	45.51 (6)	<0.001 *
		Secondary	19.1 (36)	61.7 (116)	89.4 (168)		
		Higher	17.9 (75)	64.8 (272)	91.9 (386)		
	Man	Primary or lower	41.2 (7)	52.9 (9)	47.1 (8)	26.01 (6)	<0.001 *
		Secondary	20.0 (54)	65.6 (177)	91.1 (246)		
		Higher	18.2 (61)	63.7 (214)	92.6 (311)		
Employment status	Woman	Employed	15.8 (66)	59.8 (250)	89.2 (373)	25.58 (6)	<0.001 *
		Studying	30.1 (52)	75.1 (130)	95.4 (165)		
		Unemployed	12.5 (4)	62.5 (20)	84.4 (27)		
	Man	Employed	18.5 (101)	63.4 (346)	90.7 (495)	11.53 (6)	0.073
		Studying	25.8 (16)	77.4 (48)	93.5 (58)		
		Unemployed	33.3 (5)	40.0 (6)	80.0 (12)		
Ethnicity	Woman	Bulgarian	19.4 (107)	63.9 (353)	90.8 (501)	17.99 (6)	0.006 *
		Turkish	13.8 (8)	69.0 (40)	93.1 (54)		
		Other	53.8 (7)	53.8 (7)	76.9 (10)		
	Man	Bulgarian	19.5 (107)	63.8 (351)	90.7 (499)	11.36 (6)	0.078
		Turkish	15.5 (9)	65.5 (38)	93.1 (54)		
		Other	40.0 (6)	73.3 (11)	80.0 (12)		
Residence	—	Urban	20.0 (118)	64.3 (380)	91.0 (538)	1.64 (3)	0.650
		Rural	12.5 (4)	62.5 (20)	84.4 (27)		

Values are presented as percentages and absolute frequencies, % (n), calculated within each sociodemographic subgroup. The item permitted multiple responses; therefore, percentages across the GP, school, and family categories do not sum to 100%. Overall associations were assessed using the chi-square significance test for multiple-response sets in IBM SPSS Custom Tables. Empty sociodemographic categories containing no observations were excluded. Bonferroni adjustment was applied to post hoc comparisons of column proportions within each subtable; these comparisons are not displayed. The reported χ^2^ and *p*-values represent the omnibus tests. * *p* < 0.05.

**Table 3:** Bivariate Associations of Sociodemographic Characteristics with Evaluation of the Premarital Preventive Examination, Reported Receipt of Genetic Counselling, and Perceived Necessity of Genetic Counselling.

Predictor Variable	Evaluation of Premarital Medical Examination χ^2^, *p*	Receipt of Medical-Genetic Consultant Reported Receipt of Genetic Counselling χ^2^, *p*	Perceived Necessity of Genetic Counselling Among Women, χ^2^, *p*	Perceived Necessity of Genetic Counselling Among Men, χ^2^, *p*
Ethnicity (woman)	3.203, *p* = 0.539 ^†^	14.974, *p* = 0.001 *	3.526, *p* = 0.172	4.760, *p* = 0.093
Ethnicity (man)	7.472, *p* = 0.108 ^†^	16.642, *p* < 0.001 *	2.563, *p* = 0.278	2.104, *p* = 0.349
Employment status (woman)	32.624, *p* < 0.001 *	3.733, *p* = 0.155	0.052, *p* = 0.974	0.043, *p* = 0.979
Employment status (man)	8.822, *p* = 0.061 ^†^	2.513, *p* = 0.285	1.346, *p* = 0.510	2.313, *p* = 0.315
Educational attainment (woman)	21.747, *p* < 0.001 ^†^*	5.915, *p* = 0.052	2.048, *p* = 0.359	3.748, *p* = 0.153
Educational attainment (man)	6.530, *p* = 0.158 ^†^	11.689, *p* = 0.003 *	3.061, *p* = 0.216	6.347, *p* = 0.042 *
Age group (woman)	12.395, *p* = 0.002 *	27.008, *p* < 0.001 *	0.038, *p* = 0.845	0.914, *p* = 0.339
Age group (man)	18.674, *p* < 0.001 *	17.681, *p* < 0.001 *	1.751, *p* = 0.186	5.221, *p* = 0.022 *

Pearson’s chi-square tests were used to assess bivariate associations between sociodemographic characteristics and the study outcomes. Where more than 20% of cells had expected counts below five, Monte Carlo estimates of the exact two-sided *p*-value based on 10,000 sampled tables are reported and marked with ^†^. Outcome variables represent shared couple-level responses unless otherwise specified. Associations were examined separately according to women’s and men’s sociodemographic characteristics. The analyses were exploratory. * *p* < 0.05.

**Table 4:** Multivariable logistic regression models for reported receipt of genetic counselling and affirmative support for the necessity of the premarital medical examination.

Predictor	Reported Receipt of Genetic Counselling, aOR (95% CI)	*p*	Perceived Necessity of Premarital Medical Examination, aOR (95% CI)	*p*
Female age, per one-year increase	0.929 (0.900–0.959)	<0.001	1.043 (1.006–1.081)	0.021
Higher education	1.011 (0.696–1.468)	0.955	1.284 (0.825–1.999)	0.269
Employed	0.851 (0.559–1.296)	0.453	1.847 (1.109–3.076)	0.018
Urban residence	0.911 (0.398–2.085)	0.826	0.556 (0.227–1.362)	0.199
Bulgarian ethnicity (reference: non-Bulgarian)	0.499 (0.291–0.856)	0.012	1.423 (0.780–2.597)	0.251

aOR, adjusted odds ratio; CI, confidence interval. All explanatory variables were entered simultaneously into each model. Reference categories were secondary education or lower, not employed, rural residence, and non-Bulgarian ethnicity. For the second outcome, “no” and “cannot judge” were combined and coded as 0.

## Data Availability

The data that support the findings of this study are available from the corresponding author upon reasonable request. The data are not publicly available due to privacy considerations and the protection of participants’ confidentiality.

## Abbreviations

The following abbreviations are used in this manuscript:

GP	General Practitioner
HIV	Human Immunodeficiency Virus
OB-GYN	Obstetrician–Gynaecologist
PCC	Preconception Care

## References

[1] Alswaidi F.M., O’brien S.J. (2009). Premarital screening programmes for haemoglobinopathies, HIV and hepatitis viruses: Review and factors affecting their success. J. Med. Screen..

[2] Hachmeriyan M., Levkova M., Yahya D., Stoyanova M., Dimitrova E. (2025). Genetic counseling for hereditary cancer syndromes: A 5-year experience from a single center in Bulgaria. Oncol. Rev..

[3] Zlotogora J. (2009). Population programs for the detection of couples at risk for severe monogenic genetic diseases. Hum. Genet..

[4] Maslyankov I., Hernández M. (2024). The prevalence and determinants of unmet healthcare needs in Bulgaria. PLoS ONE.

[5] Giouleka S., Papagera V., Siargkas A., Michos G., Liberis A., Kalogiannidis I., Mamopoulos A., Tsakiridis I., Dagklis T. (2025). Preconception care: A comparative review of major guidelines. Obstet. Gynecol. Surv..

[6] Scheele J., Smith S.M., Wahab R.J., Bais B., Steegers–Theunissen R.P.M., Gaillard R., Van Der Vliet-Torij H.W.H. (2023). Current preconception care practice in the Netherlands—An evaluation study among birth care professionals. Midwifery.

[7] Cristodoro M., Dell’Avanzo M., Ghio M., Lalatta F., Vena W., Lania A., Sacchi L., Bravo M., Bulfoni A., Di Simone N. (2023). Before is better: Innovative multidisciplinary preconception care in different clinical contexts. J. Clin. Med..

[8] Cassinelli E.H., McKinley M.C., Kent L., Eastwood K.-A., Schoenaker D.A.J.M., Trew D., Stoikidou T., McGowan L. (2024). Preconception health and care policies, strategies and guidelines in the UK and Ireland: A scoping review. BMC Public Health.

[9] Rechel B., Blackburn C.M., Spencer N.J., Rechel B. (2011). Regulatory barriers to equity in a health system in transition: A qualitative study in Bulgaria. BMC Health Serv. Res..

[10] Atanasova E., Panayotova S. (2024). Which factors influence health services utilization in Bulgaria? Results of a cross-sectional survey. Eur. J. Public Health.

[11] Pereza N., Terzić R., Plaseska-Karanfilska D., Miljanović O., Novaković I., Poslon Ž., Ostojić S., Peterlin B. (2022). Current state of compulsory basic and clinical courses in genetics for medical students at medical faculties in Balkan countries with Slavic languages. Front. Genet..

[12] Best S., Long J.C., Fehlberg Z., Theodorou T., Hatem S., Archibald A., Braithwaite J. (2022). The more you do it, the easier it gets: Using behaviour change theory to support health care professionals offering reproductive genetic carrier screening. Eur. J. Hum. Genet..

[13] Zhong A., Darren B., Loiseau B., He L.Q.B., Chang T., Hill J., Dimaras H. (2018). Ethical, social, and cultural issues related to clinical genetic testing and counseling in low- and middle-income countries: A systematic review. Genet. Med..

[14] Matalon D.R., Zepeda-Mendoza C.J., Aarabi M., Brown K., Fullerton S.M., Kaur S., Quintero-Rivera F., Vatta M. (2023). Clinical, technical, and environmental biases influencing equitable access to clinical genetics/genomics testing: A points to consider statement of the American College of Medical Genetics and Genomics (ACMG). Genet. Med..

[15] Van Der Pal-de Bruin K.M., Cessie S.L., Elsinga J., De Jong-Potjer L.C., Van Haeringen A., Neven A.K., Verloove-Vanhorick S.P., Assendelft P. (2008). Pre-conception counselling in primary care: Prevalence of risk factors among couples contemplating pregnancy. Paediatr. Perinat. Epidemiol..

[16] Osmanska J., Jackson A.M., Simpson J., Adamson C., Doherty D., Mamet H., Moir L., Walker N.L., Hogg D., Simpson M. (2024). Preconception counselling in women of reproductive age attending cardiology clinics in Scotland. Heart.

[17] Maas V.Y.F., Poels M., Hölscher I.M., Van Vliet-Lachotzki E.H., Franx A., Koster M.P.H. (2022). How to improve preconception care in a local setting? Views from Dutch multidisciplinary healthcare providers. Midwifery.

[18] Maas V.Y.F., Poels M., Ista E., Menge L.F., Vanden Auweele K.L.H.E., De Bie R.W.A., De Smit D.J., Van Vliet-Lachotzki E.H., Franx A., Koster M.P.H. (2022). The effect of a locally tailored intervention on the uptake of preconception care in the Netherlands: A stepped-wedge cluster randomized trial (APROPOS-II study). BMC Public Health.

[19] Poels M., Van Stel H.F., Franx A., Koster M.P.H. (2018). The effect of a local promotional campaign on preconceptional lifestyle changes and the use of preconception care. Eur. J. Contracept. Reprod. Health Care.

[20] M’hamdi H.I., Van Voorst S.F., Pinxten W., Hilhorst M.T., Steegers E.A.P. (2016). Barriers in the uptake and delivery of preconception care: Exploring the views of care providers. Matern. Child Health J..

[21] Bortolus R., Filippini F., Cipriani S., Morassutti F.R., Marchetto L., Rigotti E., Cesari E., Trevisanuto D., Parazzini F. (2025). Pregnancy, risk behaviors and adverse reproductive outcomes: Is preconception care working in Italy?. Midwifery.

[22] Cassinelli E.H., McClure A., Cairns B., Griffin S., Walton J., McKinley M.C., Woodside J.V., McGowan L. (2023). Exploring Health Behaviours, Attitudes and Beliefs of Women and Men during the Preconception and Interconception Periods: A Cross-Sectional Study of Adults on the Island of Ireland. Nutrients.

[23] Schoenaker D., Lovegrove E., Santer M., Matvienko-Sikar K., Carr H., Alwan N.A., Kubelabo L., Davies N., Godfrey K.M. (2024). Developing consensus on priorities for preconception care in the general practice setting in the UK: Study protocol. PLoS ONE.

[24] Khattak H., Tsiapakidou S., Mukhopadhyay S., Mahmood T., Cameron S., Kubba A., Merki-Feld G., Savona-Ventura C., Klanjscek J., Bitzer J. (2024). Variations in sexual and reproductive health services for the provision of comprehensive contraceptive and abortion services across Europe: A questionnaire-based study commissioned by the European Board and College of Obstetrics & Gynaecology (EBCOG) and European Society of Contraception (ESC). Eur. J. Obstet. Gynecol. Reprod. Biol..

[25] Goodfellow A., Frank J., McAteer J., Rankin J. (2017). Improving preconception health and care: A situation analysis. BMC Health Serv. Res..

[26] American Society for Reproductive Medicine, American College of Obstetricians and Gynecologists’ Committee on Gynecologic Practice (2019). Prepregnancy counseling: Committee Opinion No. 762. Fertil. Steril..

[27] (2019). ACOG Committee Opinion No. 762: Prepregnancy Counseling. Obstet. Gynecol..

[28] Arluck J.C., Mayhew A.C. (2018). Preconception care for the general OB/Gyn. Clin. Obstet. Gynecol..

[29] Caut C., Schoenaker D., McIntyre E., Steel A. (2025). Health professionals’ beliefs and attitudes towards preconception care: A systematic review. BMC Health Serv. Res..

[30] Sijpkens M.K., Van Den Hazel C.Z., Delbaere I., Tydén T., Mogilevkina I., Steegers E.A.P., Shawe J., Rosman A.N. (2019). Results of a Dutch national and subsequent international expert meeting on interconception care. J. Matern.-Fetal Neonatal Med..

[31] Goossens J., De Roose M., Van Hecke A., Goemaes R., Verhaeghe S., Beeckman D. (2018). Barriers and facilitators to the provision of preconception care by healthcare providers: A systematic review. Int. J. Nurs. Stud..

[32] Kirk E.P., Delatycki M.B., Archibald A.D., Tutty E., Caruana J., Halliday J.L., Lewis S., McClaren B.J., Newson A.J., Dive L. (2024). Nationwide, Couple-Based Genetic Carrier screening. N. Engl. J. Med..

[33] Peterlin B., Peterlin A. (2025). Carrier screening and pregnancy. Best Pract. Res. Clin. Obstet. Gynaecol..

[34] Toivonen K.I., Oinonen K.A., Duchene K.M. (2016). Preconception health behaviours: A scoping review. Prev. Med..

[35] McGowan L., Lennon-Caughey E., Chun C., McKinley M.C., Woodside J.V. (2020). Exploring preconception health beliefs amongst adults of childbearing age in the UK: A qualitative analysis. BMC Pregnancy Childbirth.

[36] Dennis C.-L., Brennenstuhl S., Brown H.K., Bell R.C., Marini F., Birken C.S. (2022). High-risk health behaviours of pregnancy-planning women and men: Is there a need for preconception care?. Midwifery.

[37] Aynalem Y.A., Paul P., Kung J.Y., Hussain A., Lassi Z., Meherali S. (2025). Understanding preconception care: A scoping review of knowledge, attitudes and practices among reproductive age individuals, healthcare workers and stakeholders in low- and middle-income countries. BMJ Open.

[38] Craig A., Mabetha K., Stephenson J., Schoenaker D., Norris S.A. (2025). Preconception health knowledge, attitudes and behavioural intentions among adults: A multi-country study. Reprod. Health.

[39] Mitchell E.W., Levis D.M., Prue C.E. (2010). Preconception Health: Awareness, planning, and communication among a sample of US men and women. Matern. Child Health J..

